# Ethylenediamine assist preparation of carbon dots with novel biomass for highly sensitive detection of levodopa†

**DOI:** 10.1039/d4ra08240k

**Published:** 2025-01-03

**Authors:** Zongmei Huang, Jing Li, Lu-Shuang Li

**Affiliations:** a Key Laboratory of Tropical Biological Resources of Ministry of Education, School of Pharmaceutical Sciences, Collaborative Innovation Center of One Health, Hainan University Haikou 570228 China lvyli@hainanu.edu.cn; b Xiangyang Central Hospital, Affiliated Hospital of Hubei University of Arts & Science Xiangyang 441021 China

## Abstract

Levodopa (l-Dopa), a precursor drug for dopamine has been widely used to treat Parkinson's disease. However, excess accumulation of l-Dopa in the body may cause movement disorders and uncontrollable emotions. Therefore, it is vital to monitor l-Dopa levels in patients. In this study, a carbon dot (CD)-based fluorescence sensing system was developed for sensitive detection of l-Dopa. The CDs were prepared using a novel biomass, *Pandanus amaryllifolius* Roxb., as a carbon source *via* a simple hydrothermal method. Interestingly, it was found that ethylenediamine doping in the preparation system increased the quantum yield of CDs, as well as their fluorescence response sensitivity to l-Dopa. After optimizing the preparation and sensing conditions, the detection limit of l-Dopa decreased from 1.54 μM to 0.05 μM. A complete methodological validation was conducted and the probe was successfully applied to the determination of l-Dopa in fetal bovine serum with excellent precision (RSD ≤ 2.99%) and recoveries of 88.50–99.71%. Overall, this work provides an effective strategy for the regulation of properties of CDs derived from biomass and an innovative method for clinical l-Dopa monitoring.

## Abstract 2

A highly sensitive sensor for levodopa detection was developed based on novel biomass-derived carbon dots.

## Graphical abstract

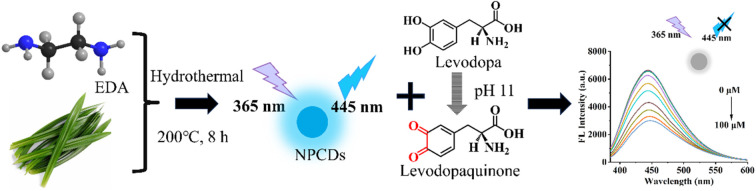

## Introduction

Parkinson's disease (PD), a common neurodegenerative disease in the elderly, is mainly caused by the lack of dopamine related to midbrain neurons. This deficiency leads to the impairment of motor functions, significantly affecting the quality of life for patients.^1^ Levodopa (l-3,4-dihydroxyphenylalanine, l-Dopa), a precursor drug for dopamine, can enter the brain through the blood–brain barrier and be converted into dopamine by dopamine-decarboxylase to supplement dopamine in the brain, thus alleviating the symptoms. l-Dopa is a commonly used clinical drug for the treatment of PD.^2^ However, long-term use of l-Dopa can increase the concentration of l-Dopa in the body, and excessive l-Dopa will lead to many side effects, such as bradykinesia, muscle stiffness, and tremors.^3^ Therefore, it is critical to monitor the concentration of l-Dopa in patients taking this drug to improve the curative effect.

To date, some traditional analytical methods for l-Dopa detection have been reported, such as high-performance liquid chromatography (HPLC), capillary electrophoresis (CE), spectrophotometric and electrochemical methods.^4,5^ These methods achieve effective detection of l-Dopa, but they have various disadvantages, including high costs, complex operation procedures, long analysis times, and easy interference. In recent years, fluorescence sensing has developed rapidly in detection of various types of targets due to its superiority of enhanced selectivity, high sensitivity, quick response, low cost, and independence from expensive instruments.^6^ Therefore, the construction of a fluorescence sensor for l-Dopa detection is a reasonable and ideal solution to overcome the limitations of traditional methods and meet clinical requirements.

Carbon dots (CDs) are a novel type of fluorescent carbon nanoparticles and have attracted much attention because of low toxicity, superior biocompatibility, high chemical stability, easy surface functionalization and minimal photo bleaching.^7^ CDs have been successfully applied in various applications, including fluorescence sensing, bioimaging, drug delivery and photocatalysis, of which fluorescence sensing is a key part.^8,9^ CDs-based fluorescence sensors mainly rely on the enhancement or quenching of fluorescence after their reaction with analytes.^10^ Surface functional groups play a major role in the reaction between carbon dots and detection objects. Hence, various CDs have been prepared in many studies by changing the carbon source or surface modification.^11,12^ These CDs have been applied in constructing fluorescence sensors for diverse analytes, including metal ions, pesticides, antibiotics and so on.^13–15^ Among them, there are few reports published on the development of CDs-based l-Dopa sensors. Therefore, there is an urgency to explore new carbon sources or preparation methods for producing CDs with high quantum yield and fluorescence properties for l-Dopa detection.

*Pandanus amaryllifolius* Roxb. is a tropical green plant common in Hainan province of China that is rich in vitamins, proteins, chlorophyll, nucleic acids, and other nutrients. Due to its low price and availability, it is an excellent precursor for preparing carbon dots. In this study, *Pandanus amaryllifolius* Roxb., was applied as the green carbon source for preparing CDs. A nitrogen-rich chemical (ethylenediamine, EDA) was deliberately introduced to the hydrothermal reaction system to regulate the properties of CDs. CDs prepared with (NPCDs) and without (PCDs) EDA were comparatively analyzed. It was found that a higher quantum yield and a more sensitive fluorescence response to l-Dopa were achieved when using NPCDs. After optimizing the preparation and detection conditions, a fluorescence sensor for l-Dopa was developed using NPCDs with the limit of detection of 0.05 μM. This sensor was successfully used to detect l-Dopa in fetal bovine serum samples with excellent precisions (RSD ≤ 2.99%) and recoveries of 88.50–99.71%. In summary, this work enriches the types of CDs and provides an innovative idea for the regulation of the properties of CDs derived from biomass carbon sources. An effective method for monitoring l-Dopa was presented, demonstrating substantial potential for clinical applications.

## Experimental

### Materials and methods

Glycine (Gly), l-alanine (l-Ala), l-cysteine (l-Cys), l-serine (l-Ser), l-threonine (l-Thr), l-leucine (l-Leu), l-glutamic (l-Glu), l-phenylalanine (l-Phe), l-tyrosine (l-Tyr) and K_2_SO_4_ were purchased from Shanghai Yien Chemical Technology Co., Ltd (Shanghai, China). Ethylenediamine (EDA), H_2_SO_4_, NaCl, NaOH, MgSO_4_ and HCl were obtained from Xilong Scientific Co., Ltd (Guangdong, China). Urea was provided by Aladdin Chemical Reagent Co. Ltd (Shanghai, China). Quinine sulfate, l-ascorbic acid (VC), β-cyclodextrin (β-CD) and dopamine (DA) were acquired from Macklin Biotech Co. Ltd (Shanghai, China). Levodopa (l-Dopa) and sodium acetate anhydrous were obtained from Shanghai Yuanye Bio-Technology Co., Ltd (Shanghai, China). Fetal bovine serum (FBS) was purchased from Zhejiang Tianhang Biotechnology Co., Ltd (Zhejiang, China). The leaves of *Pandanus amaryllifolius* Roxb. were purchased online which produced from Wanning City (Hainan, China). All solutions were prepared using ultrapure water obtained from Nova EU10 water purification system (Qingdao, China).

### Characterization

Transmission electron microscopy (TEM) images were observed on a JEM 2100F microscope (JEOL, Japan). Raman spectra were obtained by HR evolution (Horiba, France). X-ray photoelectron spectroscopy (XPS) was performed using a ESCALAB 250Xi spectrometer (Thermo Scientific, USA). Fourier Transform Infrared Spectroscopy (FTIR) spectra were recorded by a Thermo Field IS5 spectrometer (Thermo, USA). X-ray diffraction (XRD) spectra were obtained by a SmartLab-9 kW X-ray diffractometer (Rigaku, Japan). Fluorescence lifetimes were evaluated with the help of a FLS1000 fluorescence spectrometer (UK). UV-vis absorption spectra were recorded by a UV-2600 spectrophotometer (Shimadzu, Japan). Fluorescence intensities and spectra were recorded by a FL-4700 fluorescence spectrophotometer (Shimadzu, Japan). Zeta potential was measured by a ZSU310 nano-particle potential analyzer (Malvern, UK). Elemental analysis of carbon source was performed using a UNICUBE CHNSO element analyzer (Elementar, Germany). Quantum yield (QY) was measured and calculated according to previous work.^16^

### NPCDs preparation

*Pandanus amaryllifolius* Roxb. were washed, dried in the oven and ground into powder. 0.5 g of the powder, 10 mL of ultrapure water and 50 μL of ethylenediamine were mixed evenly in a 25 mL Teflon lined high-pressure reactor, and then heated at 200 °C for 8 h. After naturally cooled to room temperature, a 0.22 μm membrane was used to filter the products and thus obtain the filtrate, known as NPCDs. The filtrate was further dialyzed against ultrapure water through a dialysis bag (1000 Da molecular weight cut off) for 24 h with renewing the water every 4 h to remove the small molecules. PCDs were prepared using the same method, but without the addition of EDA in the reaction system.

### l-Dopa detection

The stock solution was prepared by dissolving l-Dopa in ultrapure water. Standard working solutions of l-Dopa with different concentrations (0.1–100 μM) were prepared by diluting the reserve solution with ultrapure water. 4 mL of working solution or samples was added to a 5 mL centrifuge tube and adjusted to pH 11 using NaOH solution. 200 μL of NPCDs solution was added. After standing for 30 minutes, the fluorescence intensity (FL intensity) of the mixture was measured at excitation and emission wavelengths of 365 nm and 440 nm, respectively. The fluorescence quenching efficiency (*F*_0_/*F*) was calculated, where *F* and *F*_0_ represent the FL intensity of NPCDs/PCDs solution with and without l-Dopa, respectively. The limit of detection (LOD) was calculated by 3*σ*/*k*, where *σ* is the standard deviation of the intercept and *k* is the slope of the regression equation.

In order to investigate the selectivity, NPCDs solution was added to the solution containing a series of ions (10 μM, Mg^2+^, K^+^, SO_4_^2−^, Na^+^, CH_3_COO^−^, Cl^−^), small molecules (10 μM, Gly, l-Ala, VC, l-Cys, l-Ser, l-Thr, l-Leu, l-Glu, l-Phe, l-Tyr, urea, β-CD) or DA (1 μM), and FL intensity was measured. The above chemicals were separately added to l-Dopa solution (10 μM) to investigate the anti-interference ability for l-Dopa detection of the sensor.

The precision and accuracy experiments were conducted with three concentration levels (5 μM, 20 μM, and 60 μM). Three analysis batches over a three-day period were conducted. Precision was assessed by calculating the variation coefficient of l-Dopa samples at each concentration level. The deviation between the measured concentration and the actual concentration of l-Dopa samples was calculated for accuracy evaluation.

### Real sample analysis

Standard working solutions of l-Dopa in FBS at different concentrations (5–100 μM) were prepared by diluting the stock solution with FBS. Samples were measured following the same procedure described in section l-Dopa detection. FBS samples spiked with l-Dopa at low (10 μM), medium (20 μM) and high (60 μM) concentrations were prepared and analyzed with the sensor. The recovery was calculated according to eqn (1),

1 Recovery (%) = (*C*_measured_/*C*_added_) × 100%

where *C*_measured_ represents the l-Dopa concentration calculated through the linear regression equation of the method, and *C*_added_ is the actual l-Dopa concentration.

## Results and discussion

### Preparation of NPCDs

In this work, NPCDs were prepared by a simple one-step hydrothermal method using *Pandanus amaryllifolius* Roxb. as the carbon source for the first time. In contrast to other reported CDs with only biomass as the precursor, EDA was introduced to the reaction system for nitrogen doping to regulate the properties of CDs.^17^ The preparation conditions were optimized, including the addition amount of EDA, reaction temperature and time to increase the QY of NPCDs (QY_NPCDs_). As shown in Fig. S1a,† QY_NPCDs_ first increased and then decreased slightly with the increase of the amount of EDA. Similar to the preparation of CDs with chemical sources, N doping also resulted in improved quantum yields of CDs with biomass as carbon precursors.^18^ With the optimal doping amount of EDA (50 μL), QY_NPCDs_ was 8.19%, which was 2.66 times higher than that without EDA doping (3.08%). To optimize the reaction temperature, the preparation was carried out at 140 °C, 160 °C, 180 °C and 200 °C. As can be seen in Fig. S1b,† QY_NPCDs_ gradually increased when increasing temperature. According to previous reports, the possible fluorescence mechanisms of CDs include quantum effect, edge structure effect, surface defect states and crosslink-enhanced emission, *etc.*^19,20^ It is possible that as the temperature increase, the degree of carbonization increase, resulting in smaller particle sizes of CDs or more surface light emitting functional groups. This phenomenon may contribute to the increase of QY. Considering convenience and safety of operation, the performances at higher temperature were not investigated, and subsequent experiments were conducted at 200 °C. According to Fig. S1c,† the formation of CDs should be basically complete after 8 hours of reaction. In summary, the optimal reaction conditions for the preparation of NPCDs are as follows: 50 μL EDA, 200 °C, and the reaction time of 8 h. Under the optimal condition, QY_NPCDs_ can reach 8.19%, which is higher than many reported CDs synthesized with biomass carbon sources.^18,21,22^ Three batches of *Pandanus amaryllifolius* Roxb. leaves were purchased and used to prepare NPCDs following the same method. The reproducibility was assessed by comparing quantum yield, UV-vis spectra, fluorescence spectra, and response to l-Dopa of the NPCDs across the batches. As shown in the Table S1 and Fig. S2,† the QYs of the three batches of NPCDs exhibited minimal differences, with their spectra showing a high level of consistency and all displaying a fluorescence response to l-Dopa. These findings indicated a high degree of reproducibility in NPCDs. The synthesis yields of CDs were calculated. The yield of NPCDs (1.27 ± 0.02%) was approximately 5 times greater than that of PCDs (0.27 ± 0.03%). This result indicated that nitrogen doping may also enhance the yield of carbon dots.

### Characterization of NPCDs

**Fig. 1:**
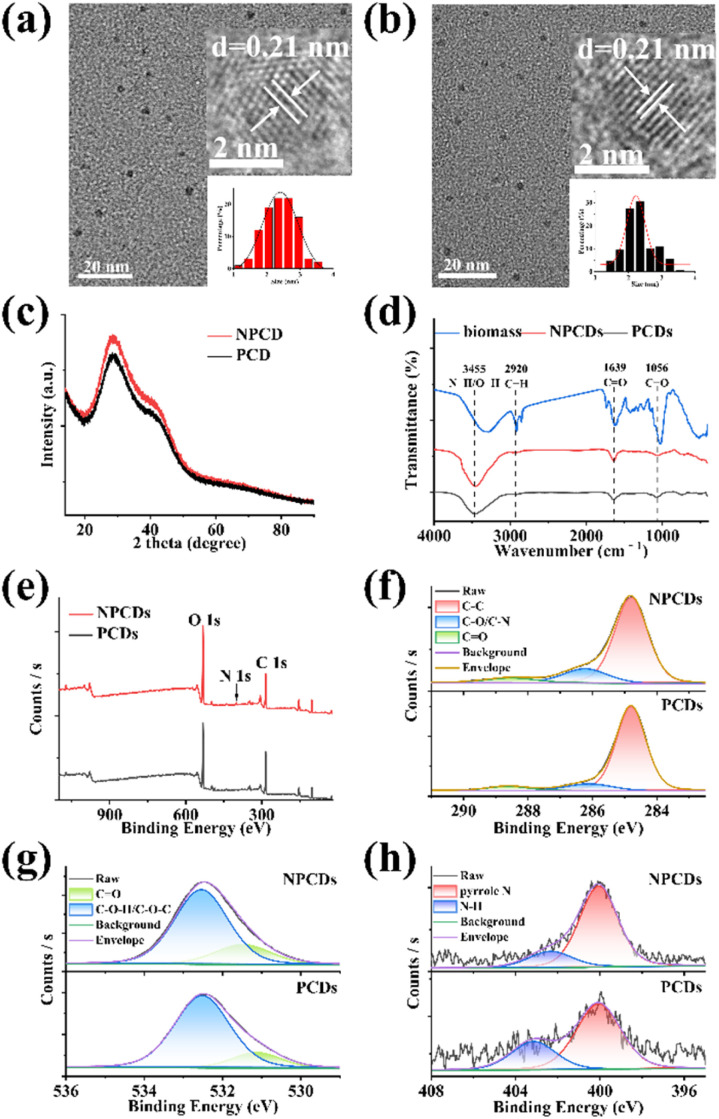
TEM images of (a) NPCDs and (b) PCDs (inset: HRTEM and size distribution of NPCDs and PCDs). (c) XRD patterns and (d) FTIR spectra of NPCDs and PCDs. (e) XPS full-scan spectrum and high-resolution XPS spectra of (f) C 1s, (g) O 1s and (h) N 1s of NPCDs and PCDs.

NPCDs and PCDs were compared to assess the impact of nitrogen doping on CDs properties. TEM images of NPCDs (Fig. 1a) and PCDs (Fig. 1b) revealed their predominantly spherical shapes with good dispersion. The diameters ranged from 1.15 nm to 3.55 nm for NPCDs and 1.45 nm to 3.55 nm for PCDs, with average sizes of 2.41 ± 0.03 nm and 2.23 ± 0.04 nm, respectively (inset of Fig. 1a and b). According to these results, nitrogen doping appeared to increase the particle size of CDs and lead to a less uniform size distribution. HRTEM images (inset of Fig. 1a and b) showed lattice spacings of 0.21 nm for NPCDs and PCDs. XRD patterns of both NPCDs and PCDs displayed diffraction peaks at approximately 29° (Fig. 1c), indicative of graphite lattice spacing (002).^18^ Both HRTEM and XRD results confirmed the graphite-like structures of NPCDs and PCDs. Raman spectra in Fig. S3† revealed D and G bands for NPCDs and PCDs, associated with sp^2^ graphitic carbon structure (ordered arrangement) and sp^3^ hybrid carbon structure (disordered arrangement), respectively.^23^ The calculated intensity ratios (*I*_D_/*I*_G_) of NPCDs and PCDs were 1.51 and 1.32, suggesting more defects presence in NPCDs compared to PCDs.^23^

FTIR and XPS were applied to explore the elements components and surface functional groups of NPCDs and PCDs. As observed in Fig. 1d, the FTIR spectra of NPCDs and PCDs exhibited similar absorption peaks at 3455 cm^−1^, 2920 cm^−1^, 1639 cm^−1^ and 1056 cm^−1^, corresponding to N–H/O–H, C–H, C=O, C–O, respectively.^24,25^ A notable difference was the stronger strength of C=O in NPCDs compared to PCDs, indicating a higher concentration of oxygen-containing groups on NPCDs. XPS survey spectra of NPCDs and PCDs revealed their predominant elements of carbon (285.0 eV), oxygen (532.3 eV), and nitrogen (400.2 eV) (Fig. 1e). The proportion of each element was calculated (Table S2†). The O/N content ratio in NPCDs was higher than in PCDs, while the C content ratio showed the opposite trend. Each element was further analyzed by high-resolution XPS. As illustrated in Fig. 1f, the high-resolution C 1s spectra of NPCDs/PCDs can be deconvoluted into three peaks at around 284.8 eV, 286.2 eV and 288.5 eV, which were attributed to C–C, C–O/C–N, and C=O, respectively.^24^ Compared to PCDs, there were more C=O and less C–O/C–N on NPCDs. In the high-resolution O 1s spectra of NPCDs/PCDs (Fig. 1g), the fitted peaks at 531.5 eV and 532.5 eV were related to the C=O and C–O–H/C–O–C groups, respectively.^25^ The content of C=O on the surface of NPCDs was obviously higher than that of PCDs. This was consistent with FTIR analysis. The high-resolution N 1s spectra of NPCDs/PCDs showed two peaks at around 400.1 eV and 402.3 eV, confirming the existence of nitrogen in the forms of pyrrole N and N–H (Fig. 1h).^25^ Overall, the FTIR and XPS analyses confirmed the presence of many hydrophilic groups such as hydroxy, carboxyl and amino groups on the surface of the prepared NPCDs and PCDs, ensuring their water solubility and modifiability. Nitrogen doping was identified as a factor influencing the elemental and functional group composition, potentially explaining the observed differences in QY.

### Optical properties of NPCDs

**Fig. 2:**
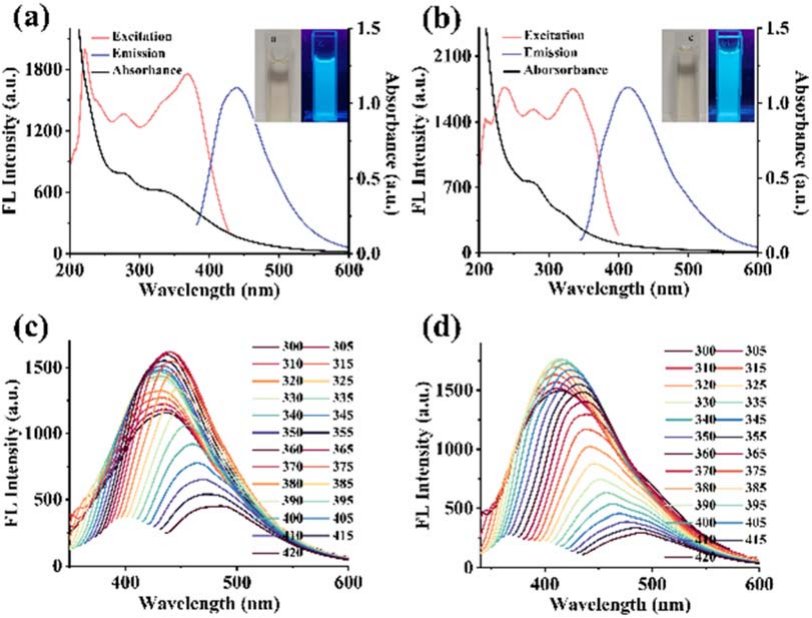
UV-vis absorption (Abs), excitation (Ex) and emission (Em) spectra of (a) NPCDs and (b) PCDs (inset: the photographs of CDs under natural light (left) and UV light of 365 nm (right)). Fluorescence emission spectra of (c) NPCDs and (d) PCDs under various excitation wavelength.

The UV-vis spectra of NPCDs (Fig. 2a) and PCDs (Fig. 2b) showed characteristic absorption peaks at 335 nm and 280 nm, attributed to n–π* transitions of C=O and π–π* transitions of C=C.^26^ There is a large difference between the UV-vis spectra of the two CDs due to the different composition of functional groups on each CDs. From the photos (inset of Fig. 2a and b), both NPCDs and PCDs appeared pale yellow in aqueous solutions under daylight and exhibited blue fluorescence under UV light (*λ* = 365 nm). PCDs and NPCDs demonstrated a wide range of wavelengths for excitation and emission (Fig. 2c and d). Besides, the fluorescence emission spectra of NPCDs and PCDs at different excitation wavelengths were examined. According to Fig. 2c and d, as the excitation wavelength increased from 300 nm to 420 nm, the fluorescence emission peaks of NPCDs and PCDs exhibited a redshift, with the fluorescence intensity initially increasing and then gradually decreasing. Both NPCDs and PCDs demonstrated excitation-dependent luminescence properties, likely due to variations in particle sizes and surface functional group compositions.^27^ The maximum excitation and emission wavelengths were used for subsequent fluorescence intensity measurements.

The impact of pH on the fluorescence of NPCDs and PCDs was investigated. As shown in Fig. S4a,† the FL intensity of NPCDs and PCDs was found to be high within a pH range of 4–11, with weak fluorescence observed in extreme acidic or alkaline conditions. In strong acid environments, there may be more free carboxyl groups on the surface of CDs, forming additional hydrogen bonds.^28^ A certain degree of aggregation of NPCDs and PCDs may result in reduced fluorescence.^28^ A strong alkaline environment will result in carboxyl group deprotonation and destroy the functional group of NPCDs and PCDs, leading to decreased fluorescence.^29^ NaCl was used to examine the effect of ionic strength on the FL intensity of NPCDs and PCDs. As can be seen in Fig. S4b,† the FL intensity of NPCDs and PCDs exhibited remarkable stability in the presence of 1.0 M NaCl, indicating excellent ionic strength stability. Moreover, prolonged exposure to 365 nm UV irradiation for 100 minutes did not significantly alter the FL intensity of NPCDs and PCDs (Fig. S4c†), highlighting their exceptional photo-stability. These findings suggest that NPCDs are a highly stable and promising material for constructing fluorescence sensors.

### l-Dopa detection

#### Optimization of sensing conditions

**Fig. 3:**
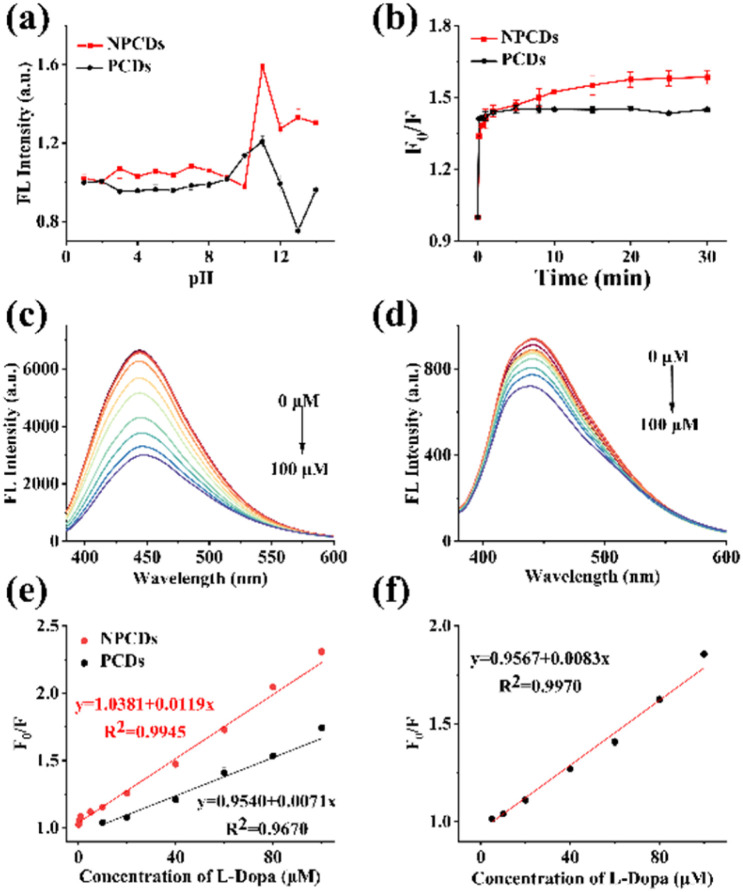
Influence of (a) pH and (b) detection time on quenching efficiency (*F*_0_/*F*). Fluorescence spectra of (c) NPCDs and (d) PCDs in the presence of l-Dopa at different concentrations. Linear relationship between *F*_0_/*F* and l-Dopa concentration in (e) ultrapure water and (f) FBS matrix.

NPCDs and PCDs were tested simultaneously to compare their sensing performance. The key sensing conditions, including pH and reaction time, were optimized to maximize detection sensitivity. It can be seen in Fig. 3a that the acidity and alkalinity of the solution had a significant influence on quenching efficiency. Notably, the fluorescence of NPCDs/PCDs was hardly quenched by l-Dopa at pH 1–8 or 13–14, with the highest quenching efficiency observed at pH = 11, which was subsequently used in the following sensing experiments. Furthermore, as depicted in Fig. 3b, the fluorescence of NPCDs and PCDs exhibited rapid quenching, reaching equilibrium after 20 minutes and 5 minutes, respectively. To streamline operations, both sensing reactions were incubated for 20 minutes.

#### Linearity and sensitivity

NPCDs and PCDs were incorporated to samples with different concentrations of l-Dopa, and their fluorescence spectra were recorded. The FL intensity of NPCDs/PCDs gradually decreased as the concentration of l-Dopa increased, while the maximum emission wavelengths remained constant, as depicted in Fig. 3c and d. A higher quenching efficiency was noted for NPCDs at equivalent l-Dopa concentrations, suggesting that NPCDs may exhibit greater sensitivity to l-Dopa. To assess their sensing capabilities, calibration curves were constructed by plotting the quenching efficiency (*F*_0_/*F*) against the l-Dopa concentration. As shown in Fig. 3e, the linear relationships were observed in the l-Dopa concentration range of 0.1–100 μM for NPCDs (*R*^2^ = 0.9945) and 10–100 μM for PCDs (*R*^2^ = 0.9670). The limits of detection (LOD) of the sensors based on NPCDs and PCDs were calculated to be 0.05 μM and 1.54 μM, respectively. NPCDs-based sensor demonstrated a lower LOD and broader calibration range. These findings suggest that N doping can enhance the sensor's sensitivity for l-Dopa detection, in addition to the QY. When compared to previously reported CDs-based sensors for l-Dopa detection (Table 1), this approach exhibits superior sensitivity and a wider linear range.

**Table 1:** Comparison of the sensor in this work with other CDs-based sensors for l-Dopa detection

Sensors	QY (%)	Response type	LOD (μM)	Linear range (μM)	Reference
GSH-CQDs	22.42	Quench	0.057	0.05–1	30
Fe-CDs	40.23	Enhance	0.102	0.85–16.95	31
CDs-TYR	38	Quench	0.09	0.3–317	32
S,N-CQDs	5.9	Quench	1.50	0–45	33
NPCDs	8.19	Quench	0.05	0.1–100	This work

The NPCDs-based fluorescence sensor was systematically validated. A total of three standard curves were measured on separate days. All standard curves demonstrated a correlation coefficient (*R*^2^) exceeding 0.99, indicating exceptional linearity. The parameters of the weighted linear regression equations were given in Table S3.† The RSD values for slope and intercept were 1.92% and 1.03%, respectively, indicating superior repeatability of the sensor.

#### Precision and accuracy

The accuracy and precision tests were conducted at three concentrations of l-Dopa (5 μM, 20 μM and 60 μM). For each concentration, three replicates were performed over three days. Table S4† displays the results, showing that the intra-day and inter-day accuracy fell within the range of 92.11% to 104.27%. Intra-day and inter-day precision were both below 8.89%. These results indicated that the method is accurate, reliable, and reproducible.

#### Selectivity and anti-interference ability

**Fig. 4:**
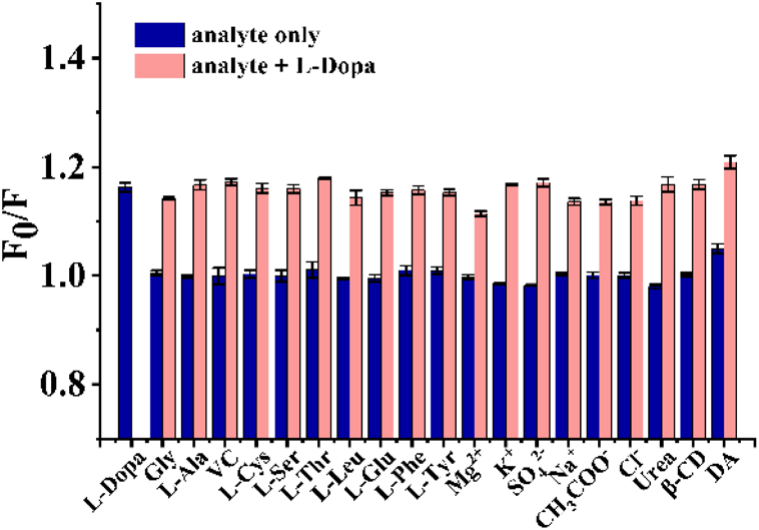
Selectivity and anti-interference ability of the sensor for various organic compounds and ions.

There are a variety of chemicals such as ions (Mg^2+^, K^+^, SO_4_^2−^, Na^+^, CH_3_COO^−^, Cl^−^), amino acids (Gly, l-Ala, l-Cys, l-Ser, l-Thr, l-Leu, l-Glu, l-Phe, l-Tyr), and other organic compounds (urea, β-CD, DA, VC) in serum that can be present alongside the target compound, l-Dopa.^30–33^ Consequently, it is essential to assess the possible interference caused by these substances. For selectivity validation, the fluorescence of NPCDs was measured in the presence of various organic compounds or ions, respectively. The quenching efficiency (*F*_0_/*F*) was calculated based on the FL intensity without (*F*_0_) and with (*F*) each substance. According to Fig. 4 (blue bar), only l-Dopa significantly quenched fluorescence of NPCDs, demonstrating the good selectivity of the sensor. When DA was introduced into the sensing system at a concentration of 1 μM, DA could partially quench the fluorescence of NPCDs, which may be through a similar quenching mechanism due to its similar catechol structure with l-Dopa. As we know, DA concentration in normal people's blood is below 130 pM.^34^ In contrast, the concentration of l-Dopa in the blood of patients following treatment may reach levels between 2.54–8.11 μM,^35^ which is more than 100 times higher than that of DA. DA therefore has a negligible effect on l-Dopa sensing in serum. The interference of coexisting substances was further investigated by adding different substances to the l-Dopa standard solution. The samples were then analyzed *via* the same procedure. As seen in Fig. 4 (pink bar), there is little effect of other coexistence chemicals on the quenching efficiency of NPCDs by l-Dopa, suggesting a good anti-interference ability of the sensor.

#### Detection of l-Dopa in real samples

The composition of FBS and human serum is similar, as both contain a variety of plasma proteins, polypeptides, fats, carbohydrates, growth factors, hormones, and inorganic substances. This similarity makes FBS a suitable substitute for blood plasma in method validation, a practice commonly utilized in previous researches and also implemented in this study.^36–38^ Standard samples were prepared by spiking l-Dopa in FBS matrix. By following the same detection process, the relationship between quenching efficiency and the concentration of l-Dopa was explored. As illustrated in Fig. 3f, a decent linearity was observed within the concentration range of 5 to 100 μM, with a correlation coefficient of 0.9970, indicating that the influence of the matrix on the detection was negligible. A spike recovery test was carried out at three concentrations. The calculated recoveries (ranging from 88.50% to 99.71%) and RSDs (ranging from 2.08% to 2.99%) further verified the accuracy of the method in real sample detection (Table S5†).

#### Sensing mechanism

**Fig. 5:**
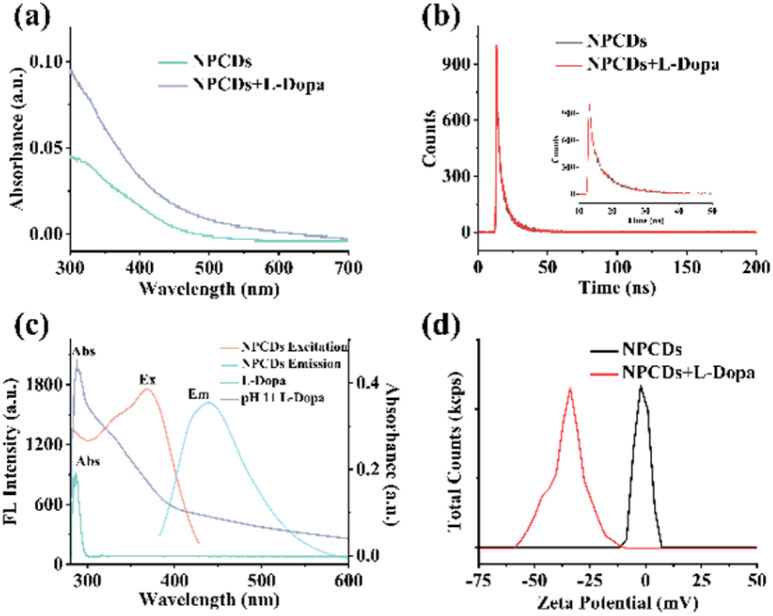
(a) UV-vis absorption spectra of the NPCDs-based sensing system with and without l-Dopa at pH 11. (b) Fluorescence lifetime measurements of NPCDs without and with l-Dopa (inset: the detail view of (b) from 10 to 50 ns). (c) UV-vis absorption spectrum of l-Dopa at neutral and at pH 11; excitation and emission spectra of NPCDs. (d) Zeta potential of NPCDs with and without l-Dopa in the system.

To explore the sensing mechanism, the Stern–Volmer equation, UV absorption spectra, fluorescence spectra, fluorescence lifetime decay curves and Zeta potential were exploited. According to Fig. 3e, the fluorescence quenching of NPCDs by l-Dopa fitted well to the Stern–Volmer equation (*F*_0_/*F* = 1 + *K*_SV_[l-Dopa]), and *K*_SV_ (Stern–Volmer constant) was calculated to be 1.72 × 10^4^ M^−1^. The fluorescence quenching may be through static or dynamic quenching mechanisms.^39,40^ UV absorption spectra of NPCDs with and without l-Dopa were examined at pH 11. In Fig. 5a, after adding l-Dopa, the absorption peaks of NPCDs did not shift and no new peaks appeared, suggesting that static quenching might not appear.^41^ Fluorescence decay curves of NPCDs with and without l-Dopa (20 μM) were measured. After fitting with the double exponential function, the average lifetimes without and with l-Dopa were calculated to be 5.51 ns and 5.50 ns, respectively (Fig. 5b). The basically unchanged fluorescence lifetime of NPCDs demonstrated that there was no dynamic quenching process and photo-induced electron transfer (PET).^42^ The absorption of l-Dopa and l-Dopa of pH 11 and fluorescence spectra of NPCDs were compared for further investigation (Fig. 5c). When the pH was adjusted to 11, the absorption of l-Dopa increased obviously in the wavelength range of 280–600 nm. This stems from its oxidation to dopaquinone under an alkaline condition.^30^ In this situation, there is a large overlap between the excitation/emission spectra of NPCDs and the absorption of l-Dopa. Thus, the quenching may occur through Förster resonance energy transfer (FRET) and internal filter effect (IFE) mechanisms. Since no change in lifetime of NPCDs was observed, the FRET mechanism was ruled out.^42^ In addition, the zeta potential of NPCDs at pH = 11 was measured to be −1.54 mV, and it changed to −36.92 mV when l-Dopa was added, further proving that l-Dopa was oxidized (Fig. 5d). Overall, the quenching effect of l-Dopa on the fluorescence of NPCDs may be mediated primarily by IFE after its oxidation into dopaquinone.

## Conclusions

In this research, a new biomass material (*Pandanus amaryllifolius* Roxb.) was utilized as a sustainable carbon source for the preparation of carbon dots (CDs). Through comparative analysis, it was found that when CDs were prepared with EDA doping (NPCDs), a higher quantum yield and a more sensitive fluorescence response to l-Dopa were achieved. By optimizing the preparation and detection conditions, a fluorescence sensor for l-Dopa detection based on NPCDs was developed, achieving a limit of detection (LOD) of 0.05 μM. The sensor was successfully applied for detecting l-Dopa in fetal bovine serum samples with promising outcomes. Compared to existing CDs-based l-Dopa sensors, the sensor in this study offers advantages such as simple preparation, high selectivity, strong anti-interference capability, and enhanced sensitivity. The findings suggest that the NPCDs-based sensor has potential for monitoring l-Dopa in clinical settings.

## Data availability

The data supporting this article have been included as part of the ESI.†

## Author contributions

Zongmei Huang: conceptualization, methodology, investigation, writing – original draft, funding acquisition. Jing Li: writing – review & editing. Lu-Shuang Li: conceptualization, supervision, data curation, funding acquisition.

## Conflicts of interest

There are no conflicts to declare.

## Supplementary Material

RA-015-D4RA08240K-s001

## References

[cit1] Santos A. M., Wong A., Ferreira L. M. C., Soares F. L. F., Fatibello-Filho O., Moraes F. C., Vicentini F. C. (2021). Electrochim. Acta.

[cit2] Beckers M., Bloem B. R., Verbeek M. M. (2022). npj Parkinson's Dis..

[cit3] Sun X., Wang N., Xie Y., Chu H. C., Wang Y., Wang Y. (2021). Talanta.

[cit4] Becker S., Schulz A., Kreyer S., Dressler J., Richter A., Helmschrodt C. (2023). Talanta.

[cit5] Wang F., Li J. H., Chen X. X., Feng H., Liao H. Y., Liu J. L., Qian D., Waterhouse G. I. N. (2024). Chem. Eng. J..

[cit6] Qu X., Wang J., Zhang R., Zhao Y., Li S., Wang Y., Liu S., Huang J., Yu J. (2020). Microchim. Acta.

[cit7] Park S. J., Park J. Y., Chung J. W., Yang H. K., Moon B. K., Yi S. S. (2020). Chem. Eng. J..

[cit8] Yang S., Zhu H. M., Cai S. H., Chen Z. F., Liang X., Li Z., Peng N. N., Wang J. M., Wang Y. Z. (2024). Talanta.

[cit9] Lin C. H., Dhenadhayalan N., Lin K. C. (2022). Mater. Today Nano.

[cit10] Yan F., Wang X., Wang Y., Yi C., Xu M., Xu J. (2022). Microchim. Acta.

[cit11] Wang W., Wu J., Xing Y., Wang Z. (2022). Sens. Actuators, B.

[cit12] Yin C., Sun Q., Wu M., Yu X., Niu N., Chen L. (2024). Sens. Actuators, B.

[cit13] Yang L., Hu W., Pei F., Liu Z., Wang J., Tong Z., Mu X., Du B., Xia M., Wang F., Liu B. (2024). Food Chem..

[cit14] Li W., Yu X., Tang Y., Li Z., Shah S. J., Liu Y., Zhao H., Song M., Li J., Wang G., Zhou L., Zhao Z., Liu S., Zhao Z. (2023). Sens. Actuators, B.

[cit15] Miao J., Ji W., Yu J., Cheng J., Huang Y., Arabi M., Zhou N., Li B., Zhang Z., Chen L., Wang X. (2023). Sens. Actuators, B.

[cit16] Li L. S., Jiao X. Y., Zhang Y., Cheng C., Huang K., Xu L. (2018). Sens. Actuators, B.

[cit17] Li Y., Li W., Yang X., Kang Y., Zhang H., Liu Y., Lei B. (2020). ACS Appl.
Nano Mater..

[cit18] Liao X., Chen C., Zhou R., Huang Q., Liang Q., Huang Z., Zhang Y., Hu H., Liang Y. (2020). Dyes Pigm..

[cit19] Yan F., Sun Z., Zhang H., Sun X., Jiang Y., Bai Z. (2019). Microchim. Acta.

[cit20] Hu X., Liu M., Yi X., Long W., Tian J., Chen L., Zhu W., Li X., Zhang X., Wei Y. (2023). Dyes Pigm..

[cit21] Zhu Z., Yang P., Li X., Luo M., Zhang W., Chen M., Zhou X. (2020). Spectrochim. Acta, Part A.

[cit22] Qi H., Liu C., Jing J., Jing T., Zhang X., Li J., Luo C., Qiu L., Li Q. (2022). Dyes Pigm..

[cit23] Zhang X., Guo H., Chen C., Quan B., Zeng Z., Xu J., Chen Z., Wang L. (2023). Appl. Mater. Today.

[cit24] Ayad M. M., Abdelghafar M. E., Torad N. L., Yamauchi Y., Amer W. A. (2023). Chemosphere.

[cit25] Esmaeili M., Wu Z., Chen D., Singh A., Sonar P., Thiel D., Li Q. (2022). Adv. Powder Technol..

[cit26] Zhang J., Zheng G., Tian Y., Zhang C., Wang Y., Liu M., Ren D., Sun H., Yu W. (2022). Chem. Commun..

[cit27] Jacinth Gracia K. D., Thavamani S. S., Amaladhas T. P., Devanesan S., Ahmed M., Kannan M. M. (2022). Chemosphere.

[cit28] Pang S., Zhang Y., Wu C., Feng S. (2016). Sens. Actuators, B.

[cit29] Tang S., Chen D., Guo G., Li X., Wang C., Li T., Wang G. (2022). Sci. Total Environ..

[cit30] Park S. W., Kim T. E., Jung Y. K. (2021). Anal. Chim. Acta.

[cit31] Ahmad H. A., Hassan R. O. (2023). Luminescence.

[cit32] Li H., Liu J., Guo S., Zhang Y., Huang H., Liu Y., Kang Z. (2015). J. Mater. Chem. B.

[cit33] Hemmati A., Emadi H., Nabavi S. R. (2023). ACS Omega.

[cit34] Sun J., Ma Z., Cai H., Di J. (2023). Colloids Surf., A.

[cit35] Cembalo G., Ciriello R., Tesoro C., Guerrieri A., Bianco G., Lelario F., Acquavia M. A., Di Capua A. (2023). Molecules.

[cit36] Qiao X.
H., Li H. M., Ma H. J., Zhang H., Jin L. H. (2023). Talanta.

[cit37] Liu B. Q., Wei S. S., Liu E. Q., Zhang H. Y., Lu P. J., Wang J. L., Sun G. Y. (2022). Spectrochim. Acta, Part A.

[cit38] Wu Z. L., Li C. K., Yu J. G., Chen X. Q. (2017). Sens. Actuators, B.

[cit39] Kaur J., Sharma S., Mehta S. K., Kansal S. K. (2020). Spectrochim. Acta, Part A.

[cit40] Sonthanasamy R. S. A., Sulaiman N. M. N., Tan L. L., Lazim A. M. (2019). Spectrochim. Acta, Part A.

[cit41] Zhong Y. J., Chen A. L., Yin X. H., Li R. J., Deng Q. F., Yang R. (2023). Sens. Actuators, B.

[cit42] Zu F. L., Yan F. Y., Bai Z. J., Xu J. X., Wang Y. Y., Huang Y. C., Zhou X. G. (2017). Microchim. Acta.

