# Supplementary material for: Ethylenediamine assist preparation of carbon dots with novel biomass for highly sensitive detection of levodopa
Source: RSC Adv. 2025 Jan 3;15(1):420–7. doi: 10.1039/d4ra08240k (PMC11697296; doi:10.1039/d4ra08240k)
Supplement: RA-015-D4RA08240K-s001 [file RA-015-D4RA08240K-s001.pdf]

Supplementary Information (SI) for RSC Advances.
This journal is © The Royal Society of Chemistry 2024

Supplementary material

Ethylenediamine assist preparation of carbon dots with novel biomass for highly sensitive detection of levodopa

Zongmei Huang,[a] Jing Li[b] and Lu-Shuang Li*[a]

[a] Key Laboratory of Tropical Biological Resources of Ministry of Education, School of Pharmaceutical Sciences, Collaborative Innovation Center of One Health, Hainan University, Haikou 570228, China.

[b] Xiangyang Central Hospital, affiliated hospital of Hubei University of Arts & Science, Xiangyang 441021, China.

*Corresponding Author

E-mail address: lvyli@hainanu.edu.cn (L.-S. Li)

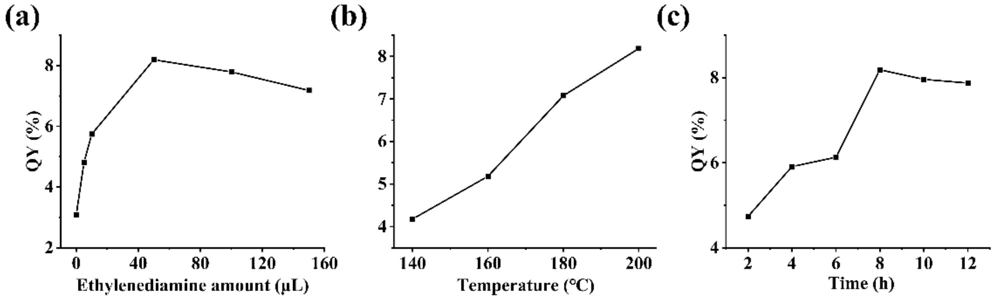

Fig. S1. QY of NPCDs prepared with different amounts of EDA (a) at different reaction temperatures (b) and of different reaction time (c).

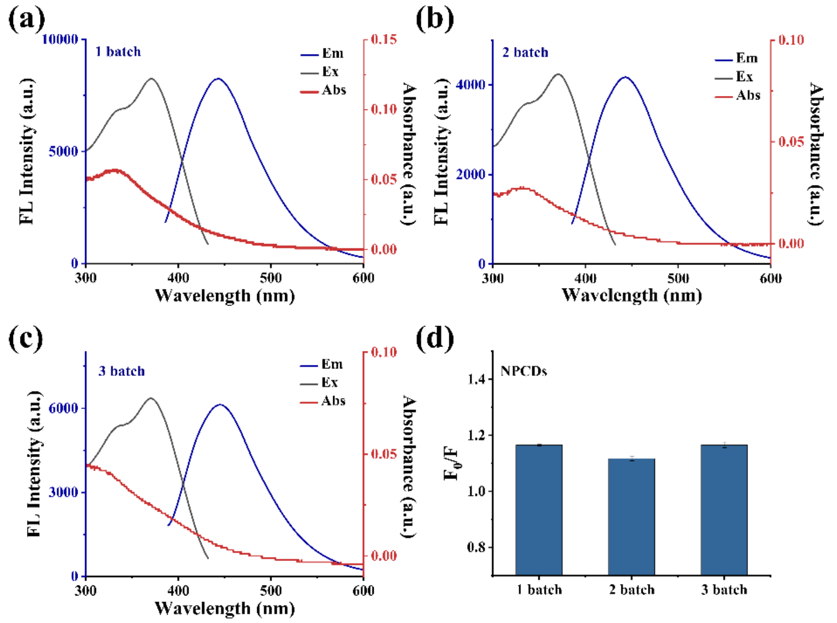

Fig. S2. UV-vis absorption (Abs), excitation (Ex) and emission (Em) spectra of NPCDs of first (a), second (b) and third batch (c). (d) The fluorescence response (F_0/F) of different batches of NPCDs towards L-Dopa at 10 μM.

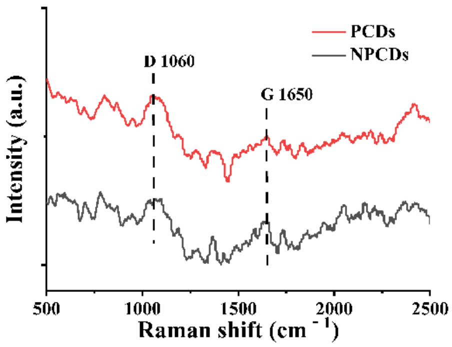

Fig. S3. Raman spectra of NPCDs and PCDs.

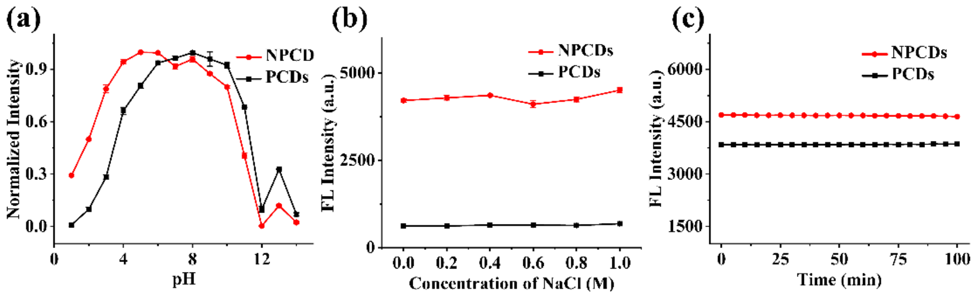

Fig. S4. (a) The normalized fluorescence spectra of NPCDs and PCDs at different pH. Fluorescence intensity of NPCDs and PCDs (b) at different NaCl concentrations and (c) different time under continuous UV irradiation.

Table S1 The QYs of three batches of NPCDs.

Batch	QY (%)
1	8.18
2	7.96
3	8.19

Table S2 The content of C, O and N elements in NPCDs and PCDs.

CDs	Content (%)		
	C	O	N
NPCDs	50.51	47.53	1.96
PCDs	58.26	40.82	0.91
Biomass	42.20	50.11	2.02

Table S3 The parameters of regression equations of calibration curves for different 3 days.

Run	Slope	Intercept	R^2
1	0.0119	1.0381	0.9945
2	0.0123	1.0242	0.9930
3	0.0119	1.0173	0.9938
Mean	0.0120	1.0265	0.9938
RSD (%)	1.92	1.03	−

Table S4 Intra-day and inter-day accuracy and precision of the sensor in the detection of L-Dopa. Samples were prepared on same day (n=3) for intra-day assessments, and 3 different days (n=3) for inter-day assessments.

Conc. (μM)	Intra-day			Inter-day		
	Mean ± SD	Accuracy (%)	Precision (%)	Mean ± SD	Accuracy (%)	Precision (%)
5	4.61 ± 0.11	92.11	2.40	5.21 ± 0.46	104.27	8.89
20	19.37 ± 0.21	96.83	1.06	19.14 ± 0.60	95.69	3.12

60	57.18 ± 1.07	95.31	1.88	59.15 ± 1.80	98.58	3.05

Table S5 Detection of L-Dopa in FBS.

Sample	Added (μM)	Measured (μM)	RSD (%)	Recovery (%)
1	10	9.97	2.99	99.71
2	20	19.70	2.08	98.50
3	60	53.10	2.30	88.50
